# Railway traffic characterisation data based on weigh-in-motion and machine learning: A case study in Portugal

**DOI:** 10.1016/j.dib.2025.111872

**Published:** 2025-07-11

**Authors:** Idilson A. Nhamage, Cláudio S. Horas, José A. Campos e Matos, João Poças Martins

**Affiliations:** a CONSTRUCT-LESE, University of Porto, Faculty of Engineering, 4200-465 Porto, Portugal; b ISISE, University of Minho, School of Engineering, 4804-533 Guimarães, Portugal; c CONSTRUCT- GEQUALTEC, University of Porto, Faculty of Engineering, 4200-465 Porto, Portugal

**Keywords:** Railway traffic data, Train classification, Ageing railway bridges, Structural integrity, Fatigue

## Abstract

Metallic railway bridges built during the 20th century are ageing, but their continued operation is essential for enhancing the capacity of the network. Structural degradation phenomena are a cause for concern, particularly fatigue, which requires accurate knowledge of service railway loads for structural integrity assessment in order to avoid unnecessary strengthening or premature bridge replacement. Quantifying present traffic scenarios is, therefore, critical and provides a basis for deriving past and future scenarios, given the challenges of implementing permanent weighing systems. Weigh-In-Motion (WIM) approaches are used to periodically collect data on train loads and geometry, generating large datasets containing information such as axle loads, axle spacings, and train speeds. However, identifying train types in these data is often a manual and laborious task, with this information being crucial not only for structural assessment, such as fatigue analysis, but also for broader considerations within the railway sector, including economic and social impacts. This paper presents the outcomes from a WIM system installed on the Alcácer do Sal bridge in Portugal to capture real-time train data, which was then post-processed through an automated Machine Learning (ML) approach for train classification. The resulting information is valuable not only for the national context but also for other countries with comparable traffic characteristics.

Specifications Table

**Table:** 

Subject	Railway engineering.
Specific subject area	Railway traffic characterisation through weigh-in-motion enhanced by machine learning.
Type of data	Table. Analysed, Processed.
Data collection	Railway traffic data includes train speeds, axle spacings, and axle loads, along with train type classification for the vehicles operating on the target railway line. Train speed, axle spacings, and axle loads are measured using Weigh-In-Motion (WIM) technology. Speed and axle spacings are determined using instrumented rail pads with fibre optic sensors (FORPS-UIC60–1–20), while axle loads are derived from rail shear measurements using strain gauges in a Wheatstone bridge. Train type classification is achieved by deriving Machine Learning (ML) models from a sub-dataset of the collected data.
Data source location	Faculty of Engineering, University of Porto, Rua Dr. Roberto Frias, 4200–465, Porto, Portugal.
Data accessibility	Repository name: Zenodo Data identification number: 10.5281/zenodo.14212706 Direct URL to data: https://doi.org/10.5281/zenodo.14212707
Related research article	None.

## 1 Value of the Data

• Using real train data, including train speed, axle spacings, and axle loads, railway management bodies can make performance-based decisions regarding infrastructure subjected to similar traffic conditions, optimising interventions while minimising costs.
• This information set is rare, particularly as open-access, reducing the need for laborious experimental work and respective classification post-processing, while introducing an innovative Machine Learning (ML) approach for train type identification.
• The scientific community can reuse these data as a foundation to develop practical tools for management bodies, such as predictive maintenance systems and traffic solutions to enhance operational efficiency.
• Information derived from these data can provide insights for broader studies, such as economic and environmental research, assisting in the optimisation of railway operations and the shaping of transport infrastructure policies and their social impact.

## 2 Background

The compilation of the presented data aims to support the structural integrity assessment and management of ageing railway bridges by providing accurate information on railway traffic. As many of these ageing metallic structures have exceeded or are about to exceed their design lifespan but are still in use, understanding the impact of train loads and other train characteristics is crucial for ensuring safety, including fatigue effects, which are particularly prevalent on such bridges. These data were collected for passing trains, including train speeds, axle spacings, axle loads, and train types circulating on the target line on the Alcácer do Sal Bridge in Portugal. The presented information was gathered and generated in two steps, namely: i) a Weigh-In-Motion (WIM) system was adopted to capture real-time train speed, axle spacings, and axle loads; and ii) train types were determined through automatic ML-based classification. Train speed and axle spacings were measured using instrumented rail pads with Fibre Optic Rail Pad Sensors (FORPS-UIC60–1–20), while axle loads were obtained using strain gauges in a Wheatstone bridge. ML approaches were implemented for faster and reliable train classification by testing three classification algorithms and selecting the most effective one based on performance.

## 3 Data Description

**Fig. 1:**
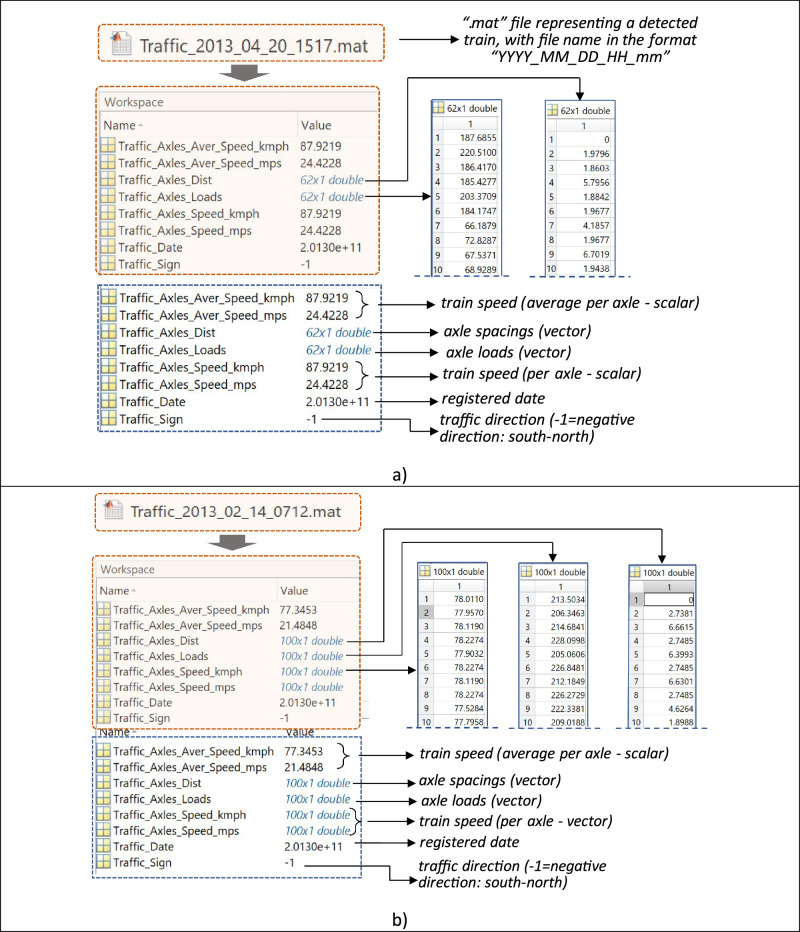
Train representation - file format and associated data: a) data with all train speed values​​ represented as scalars; and b) data with the average train axle speed represented as a scalar, alongside the non-averaged train axle speeds represented as vectors.

**Fig. 2:**
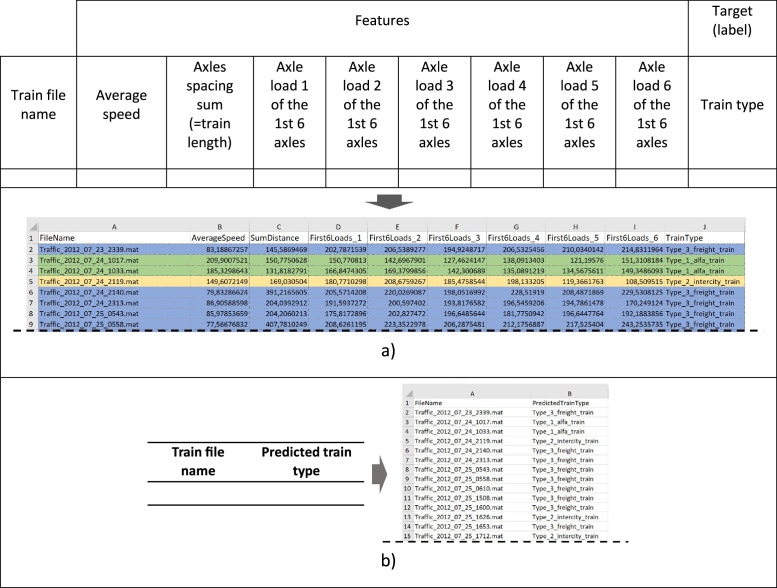
Trains classification by type: a) data format used for machine learning model training, testing and validation; and b) Output data format – train classification by type.

A Weigh-In-Motion (WIM) system was implemented to characterise the railway traffic on the Alcácer do Sal Bridge in Portugal during 2012–2013, with the resulting data stored in MATLAB files (“.mat”), according to the architecture of the experimental setup. Despite interruptions in data collection due to operational challenges, 565 trains were recorded and processed during the campaign, representing the equivalent of two months of monitoring. These data were collected mainly between late July and April, covering a range of seasons. Although the majority of the data is from August and September 2012, these months are considered to represent typical traffic trends on this line. These traffic mixes represent a total load of 0.52 million tonnes, which corresponds to an estimated annual traffic volume of 3.1 million tonnes (3.1 M tons/year). Each “. mat” file represents a detected train, containing information about train speed, axle spacings, and axle loads. A summary of the 565 registered trains during the monitoring period is given in Table 1. An example of how the data is represented in “. mat” files is shown in Fig. 1. Supporting documentation on the MAT-file format can be found in [1] . For control of data, the speed of all axles was recorded for certain trains, allowing the calculation of average speeds afterwards. In other cases, only the final average result was provided. The dataset includes an Excel file (“.xlsx”) containing trains classification by type, considering the trains circulating on the target line (Fig. 2). Overall, there are three types of trains circulating on the target route: i) Passenger train 1 – “Alfa pendular”; ii) Passenger train 2 – “Intercity”; and iii) Freight train. The data was compressed in a zip file named “Real_traffic_data” (with 856 Kilobytes) and comprises one folder and one Excel file, as follows: i) a folder with the name “Trains_set” containing MATLAB data files; and ii) an Excel file with the name “Train_classification” containing the classification of trains by type.

**Table 1:** Structure of recorded data in “.mat” files for each passing train.

#	File
1	Traffic_2012_07_23_2339.mat
2	Traffic_2012_07_24_1017.mat
3	Traffic_2012_07_24_1033.mat
(…)	
564	Traffic_2013_03_22_0716.mat
565	Traffic_2013_04_20_1517.mat

As mentioned in section 2 and detailed further in section 4, while the speed, axle spacings, and axle loads were computed using WIM data, the train classification data was achieved through ML classification algorithms. Random Forest, Logistic Regression, and Gradient Boosting-based models were tested and the most effective method was selected based on performance.

## 4 Experimental Design, Materials and Methods

### 4.1 WIM technology for measuring train speed, axle spacings and axle loads

#### 4.1.1 Overview

The Weigh-In-Motion (WIM) system has become essential in railway applications, enabling the accurate estimation of train characteristics, such as geometry and loads by analysing measurements derived directly from the railway track [2,3]. When bridge responses are considered, following a calibration process, the system is referred to as Bridge Weigh-In-Motion (B-WIM) [[4], [5], [6]]. In the case of ageing metallic railway bridges still in service, many of which were built with outdated materials and are now subjected to loads significantly different from those for which they were designed [7,8], WIM/B-WIM systems are critical in order to consider accurate traffic loading in structural integrity assessment, supporting the implementation of measures that increase structure longevity, including strengthening while optimising costs. Railway infrastructure managers are increasingly interested in using digital twin models to trigger alerts when specific damage exceeds a predefined limit, with accurate traffic loading, such as that presented, being critical for this approach [9]. In addition to structural integrity assessment, the railway traffic data acquired through WIM systems may serve for broader studies, such as economic and social impacts of railway transport.

**Fig. 3:**
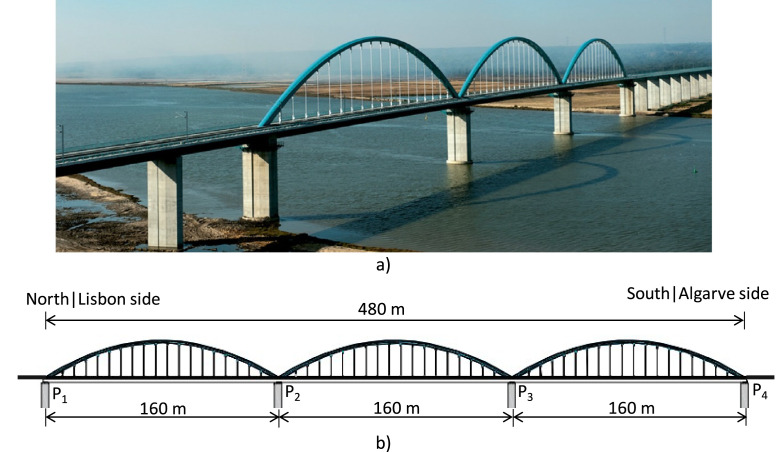
Alcácer do Sal Bridge: a) General view (photo) [12]; and b) Lateral view (schematic drawing) (adapted from Albuquerque et al. [10]).

As noted, the data in this paper is obtained from the WIM system implemented on a railway section of the Alcácer do Sal Bridge in Portugal (Fig. 3), which allows deriving conclusions about fatigue analysis in large structures using real train loadings, despite it being a relatively new structure [10,11]. This bridge is a composite bowstring-arch bridge with three continuous spans of 160 m each, a total of 480 m. Connected to it are the 1115 m North access viaduct and the 1140 m South access viaduct.

Several parameters can be considered within WIM technologies for railway traffic measurement, including: i) instrumented rail pads; ii) rail shear measurements using shear strain gauges welded or bonded to the neutral axis of the rail; iii) rail shear measurements, achieved by means of a circular slot drilled on the neutral line of the rail; and iv) rail bending measurements [12]. In this article, the first approach was implemented to obtain the speed and axle spacing of trains, while the second was used to determine axle loads.

#### 4.1.2 WIM implemented on the alcácer do sal bridge

**Fig. 4:**
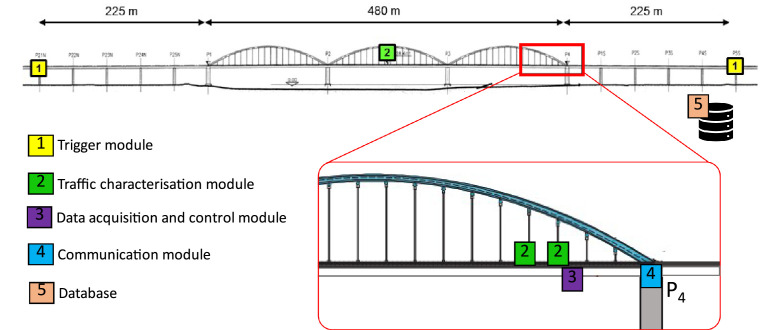
Components of the system and their location [12].

The WIM-based railway monitoring system is illustrated in Fig. 4 and consists of: i) a trigger module, which stores data when a traffic event is detected; ii) a traffic characterisation module; iii) a data acquisition and control module; iv) a communication module; and v) a database.

In the following subsections, each element of the system will be described in greater detail.

##### 4.1.2.1 Trigger module

Two uniaxial accelerometers are positioned on the access viaducts, 225 m from either end of the bridge, to initiate data recording for each traffic event. When a predefined acceleration threshold is reached, all strain gauges installed are automatically triggered, and data logging begins. The trigger threshold was established through a calibration process involving the analysis of acceleration time-history data from a representative sample of train passages. The threshold value chosen ensured that the system would be activated only by accelerations associated with actual railway traffic, while remaining above levels generally caused by ambient influences. This approach ensured reliable traffic event identification while minimising false triggers.

##### 4.1.2.2 Traffic characterisation module

**Fig. 5:**
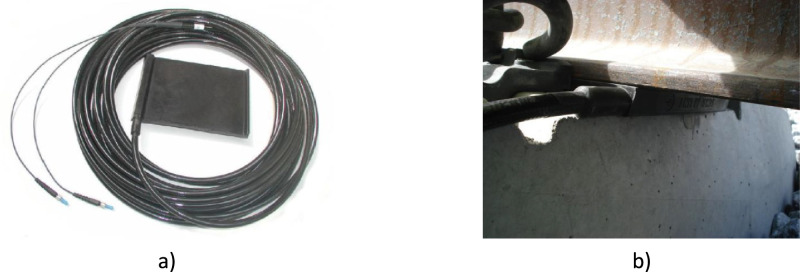
Rail pad sensors (Fibre optic rail pad sensors – FORPS): a) sensor overview; and b) sensor in place [12].

**Fig. 6:**
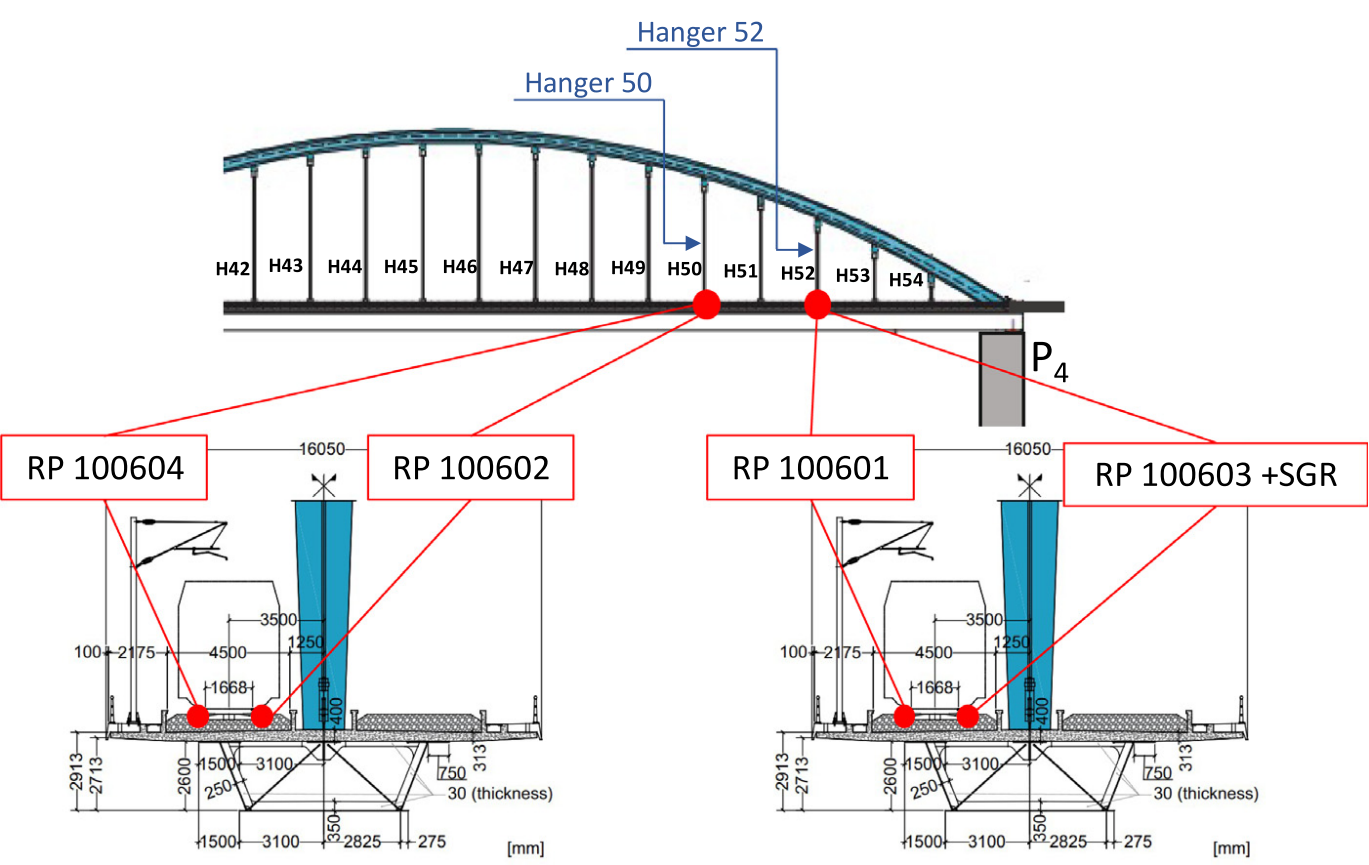
Schematic layout instrumented rail pads and strain gauges along the rail [12].

This module is formed by two parts: i) instrumented rail pads to compute the train speed and axle spacing; and ii) axle load characterisation achieved through rail shear measurements using shear strain gauges installed along the rail axis. For i), four instrumented rail pad sensors (Sensor Line FORPS-UIC60–1–20, Fig. 5) were fixed on two sleepers, spaced 16 m apart, within the same bridge section at arch hangers 50 and 52 (see Fig. 6).

These sensors are fibre optic-based and measure the strain-induced reduction of light transmission within the optical fibre embedded in the pad. Train speed and axle spacings were computed by measuring the time each axle takes to move between the two rail pad sections 16 m apart.

**Fig. 7:**
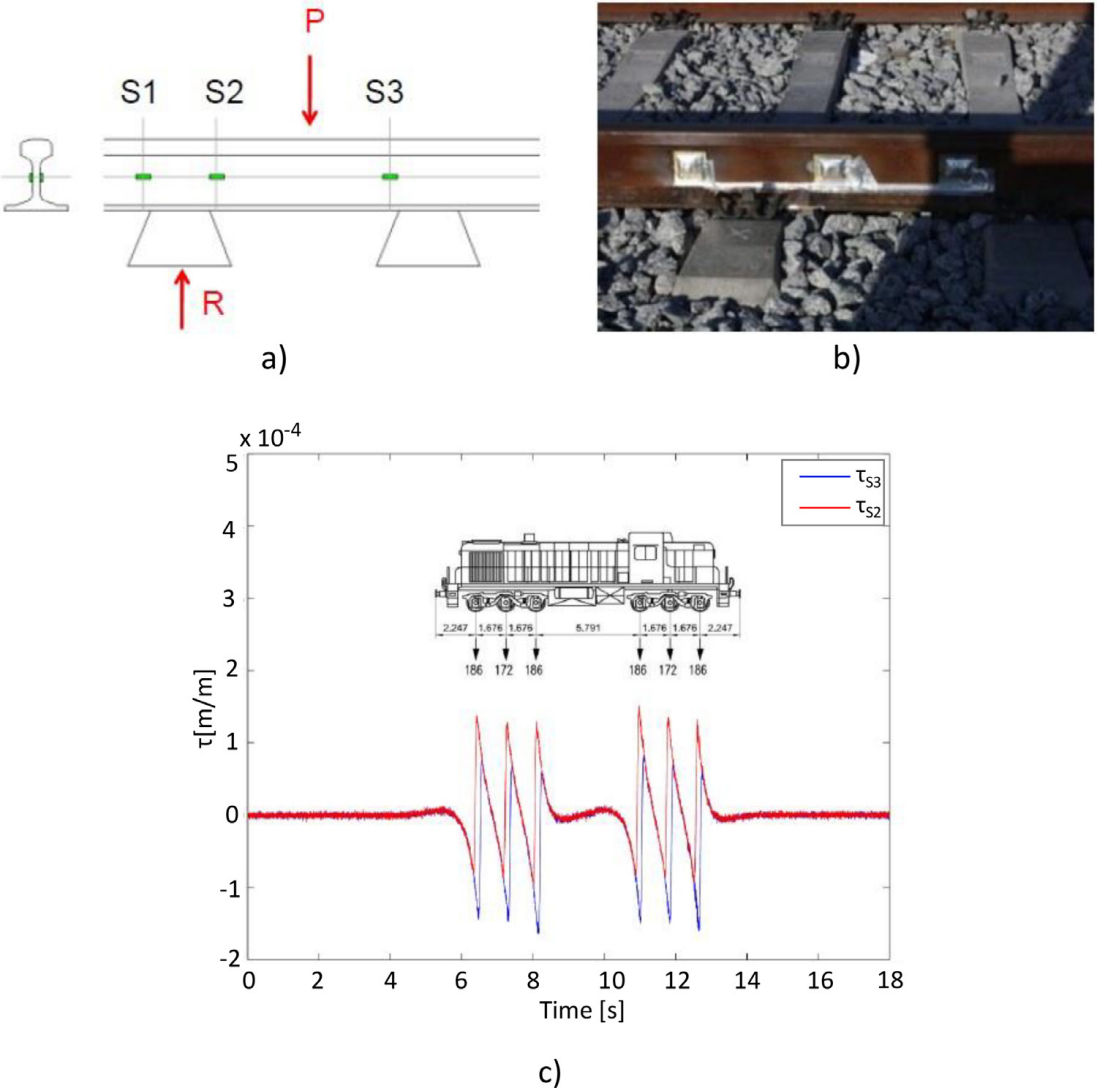
Strain gauges installation on the rail and respective signals: a) strain gauges location (schematic drawing); b) strain gauges fixed and protected (photo); and c) example of measured τ_s2_ and τ_s3_ [12].

For ii), axle loads were determined using three full Wheatstone bridges installed at different rail sections near hanger 52 (see Fig. 6). Each full Wheatstone bridge was assembled with four shear strain gauges (VISHAY LEA-06-W125F-350/3R) welded to the neutral axis of the rail. This configuration was chosen to maximise the sensitivity of the full Wheatstone bridge to vertical-plane shear stresses. Continuous measurements of shear strain were conducted at two consecutive rail sections, designated τ_s2_ and τ_s3_ (see Fig. 7a and b). The vertical load applied (F) between these sections is directly proportional to the difference in shear strain, Δτ=τ_s3_-τ_s3_ (see Fig. 7c). As stated in [13], this proportionality can be expressed by the following Eq. (1):

(1) F=Δτ·C

The constant C is determined through calibration with reference vehicles with known axle loads. For the data in this article, the value *C* = 0.707 was adopted, with F in [kN] and |Δτ| in [10^−6^ m/m].

##### 4.1.2.3 Data acquisition and control module, communication module, and database

The data acquisition and control system were based on a National Instruments - cRIO-9024 paired with an NI 9116 chassis. The chassis included several modules for interfacing with sensors and devices, namely: the NI 9236 module (for quarter-bridge strain gauges installed in the deck), the NI 9237 module (for full Wheatstone bridge strain gauges installed in the rail), and the NI 9205 module (for analog input from the instrumented rail pad transducers) [12]. A router was used to allow communication of the cRIO-9024 with a 3 G connection. This 3 G network transmitted the recorded data to a remote location, where it was subsequently saved to a network hard drive installed at the bridge location.

A database was used to store relevant information from the monitoring system logs. A MATLAB-based routine was implemented to track traffic events. As a train approached the bridge, vibration on the viaduct triggered the system, which recorded the date and time as a unique index in the format “YYYY_MM_DD_HH_mm”. Following the trigger, data from all sensors was recorded for a duration of 120 s. During this process, information to obtain the train speed, axle spacing and axle load was automatically saved.

### 4.2 Machine learning-based approach for train classification

When combined with other parameters, train type identification completes the characterisation of railway traffic and provides valuable insights for further analysis, even for non-specialists or studies that focus solely on train quantities by type or category. However, while the WIM system allows for real-time measuring of the train speed, axle spacings, and axle loads data, the type of vehicle associated with these data has been identified manually. This labour-intensive and time-consuming task limits the full potential of real-time data acquisition. To overcome this limitation, a supervised machine learning (ML) classification approach was explored to automate the process, enabling not only the real-time determination of train speed, axle spacings, and axle loads but also the identification of corresponding train types. As mentioned, Random Forest, Logistic Regression, and Gradient Boosting classification algorithms were tested, and the best-performing algorithm was ultimately chosen. The performance of the model was measured in terms of accuracy, precision, recall, and F1-score metrics.

Among the various types of trains operating in Portugal, three of them move across the Alcácer do Sal Bridge, namely: i) the Alfa Pendular passenger train; ii) the Intercity passenger train; and iii) freight trains of different configurations. As noted, the objective was to find the train type based on the real-time data inputs of speed, axle spacings, and axle loads, framing the task as a multi-class classification problem from a ML perspective.

Out of the 565 recorded trains (dataset), each expressed by a MATLAB file (“. mat”), as outlined in Table 1, a sub dataset (labelled dataset) consisting of the first 100 trains was selected to train the ML-based classification model. A summary of the process carried out is presented as follows.

A. Sub dataset (Table 2):

• Features: 8 features related to train speed, axles spacings and axles loads were considered, 6 of which as “sub-features” of the major “axle loads”. The first 2 features were “AverageSpeed” and “SumDistance” (which is the sum of all axle spacings, providing the train length). The remaining 6 features were the loads of the first 6 axles (“First6Loads_1, First6Loads_2, First6Loads_3, … First6Loads_6”).
• Labels: 3 class labels were defined, corresponding to the types of trains operating in Portugal that circulate on the target line, i.e., Class 1 - “Type_1_alfa_train”; Class 2 - “Type_2_intercity_train”; and Class 3 – “Type_3_freight_train ”.

**Table 2:** Sub dataset representation according to the defined features and labels.

Instance		Features						Class
#	Train file	AverageSpeed [km/h]	SumDistance [m]	First6Loads_1 [kN]	First6Loads_2 [kN]	(…)	First6Loads_6 [kN]	TrainType
1	Traffic_2012_07_23_2339_Final.mat	83.189	145.59	202.79	206.54		214.83	Type_3_freight_train
2	Traffic_2012_07_24_1017_Final.mat	209.90	150.78	150.77	142.70		151.31	Type_1_alfa_train
3	Traffic_2012_07_24_1033_Final.mat	185.33	131.82	166.85	169.38		149.35	Type_1_alfa_train
4	Traffic_2012_07_24_2119_Final.mat	149.61	169.03	180.77	208.68		108.51	Type_2_intercity_train
5	Traffic_2012_07_24_2140_Final.mat	79.83	391.22	205.57	220.03		229.53	Type_3_freight_train
6	Traffic_2012_07_24_2313_Final.mat	86.91	204.04	191.59	200.60		170.25	Type_3_freight_train
(…)								
100	Traffic_2012_07_30_1022_Final.mat	209.87	150.69	145.42	158.61		139.63	Type_1_alfa_train

The preparation of the subset involved a manual process to match the features with their corresponding labels, a step that will not need to be repeated for future traffic measurements on this bridge.

B. Sub dataset Split:

The sub dataset was divided into 65 % for training, 15 % for validation, and 20 % for testing.

C. Performance:

As shown in Table 3, 4 metrics were considered to assess the performance of the algorithms, namely:

i) Accuracy (ratio of correctly predicted instances, both true positives and true negatives to the total number of instances);
ii) Precision (ratio of correctly predicted positive observations to the total predicted positive observations);
iii) Recall (ratio of correctly predicted positive observations to all actual positive observations); and,
iv) F1-score (the harmonic mean of precision and recall, which provides a balance between the two). To assess the robustness of the model and explore feasible simplifications, a sensitivity analysis was also performed to determine the impact of reducing the number of axle load features. In addition to the 8 feature case (Case 1, C1), three more cases were evaluated:
  • Case 2 (C2): 7 features (“AverageSpeed”, “SumDistance”, and first five axle loads);
  • Case 3 (C3): 6 features (“AverageSpeed”, “SumDistance”, and first four axle loads); and,
  • Case 4 (C4): 5 features (“AverageSpeed”, “SumDistance”, and first three axle loads).

**Table 3:** Performance of train classification: metrics for different feature subsets (Cases 1 to 4).

Algorithm	Accuracy				Precision				Recall				F1 - Score			
	C1	C2	C3	C4	C1	C2	C3	C4	C1	C2	C3	C4	C1	C2	C3	C4
Logistic Regression	Validation															
	0.73	0.69	0.67	0.64	0.33	0.31	0.30	0.29	0.73	0.69	0.66	0.64	0.84	0.57	0.58	0.51
	Test															
	0.60	0.54	0.51	0.46	0.33	0.31	0.30	0.26	0.60	0.55	0.52	0.46	0.75	0.46	0.43	0.35
Random Forest	Validation															
	1.00	0.97	0.93	0.92	1.00	0.97	0.93	0.89	1.00	1.00	0.97	0.90	1.00	0.98	0.95	0.90
	Test															
	1.00	0.97	0.95	0.92	1.00	0.95	0.94	0.90	1.00	0.94	0.83	0.81	1.00	0.95	0.88	0.88
Gradient Boosting	Validation															
	0.91	0.85	0.80	0.75	0.96	0.92	0.88	0.84	0.83	0.81	0.77	0.74	0.87	0.88	0.84	0.79
	Test															
	1.00	0.96	0.95	0.93	1.00	1.00	0.97	0.95	1.00	1.00	0.95	0.84	1.00	1.00	0.96	0.92

**Table 4:** Sensitivity analysis on training sample size (Random Forest, 8 features).

No. of Training samples	20	40	60	80	85	88	90	92	95	100
Accuracy	0.05	0.15	0.90	0.95	0.98	1.00	1.00	1.00	1.00	1.00

The results demonstrated that Random Forest outperformed Logistic Regression and Gradient Boosting across all metrics, except for some metrics in the test set involving Cases 2 to 4. This made Random Forest the chosen algorithm for train type classification. While Random Forest achieved 100 % accuracy with all 8 features, performance dropped slightly as fewer axle load measurements were used. Nonetheless, Random Forest maintained great accuracy for the validation set (≥ 90 %) when only the first three axle loads were used. These findings imply that a smaller number of features could be used with minimal impact on classification performance, potentially simplifying future real-time applications.

A sensitivity analysis was also conducted to establish the minimal number of training samples required for optimal accuracy. As seen in Table 4, with 88 samples, the Random Forest classifier achieved 100 % accuracy. Hence, the choice of 100 training samples is a conservative way to ensure model reliability. However, good results might have been achieved with slightly fewer samples (nearly 90). In addition, a summary of the number of trains of each type identified using the Random Forest algorithm (the most accurate method), trained with 100 samples and using all eight features (Case 1), is presented in Table 5. Out of the 565 trains, 76 were identified as Alfa Pendular passenger trains (0.15 million tons/year), 122 as Intercity passenger trains (0.27 million tons/year), and 367 as Freight trains (2.69 million tons/year).

**Table 5:** Train types identified using Random Forest (Case 1).

Train type	No. of trains identified	Equivalent annual tonnage (10^6^ tons)
Alfa Pendular passenger train	76	0.15
Intercity passenger train	122	0.27
Freight train	367	2.69
Total	565	3.11

## Limitations

None.

## Ethics Statement

The authors affirm that they followed the ethical requirements for publication in Data in Brief and that this work does not involve human subjects, animal experiments, or the use of data from social media platforms.

## CRediT Author Statement

**Idilson A. Nhamage:** Conceptualisation, Methodology, Investigation, Software, Data curation, Writing- Original draft preparation. **Cláudio S. Horas:** Conceptualisation, Methodology, Software, Data curation, Writing- Reviewing and Editing, Supervision. **João Poças Martins:** Conceptualisation, Writing- Reviewing and Editing, Supervision. **José A. Campos e Matos:** Supervision.

## Data Availability

ZenodoReal railway traffic - Alcacer Bridge in Portugal (Original data)
